# The effects of formulated palmitoylethanolamide supplementation on indicators of stress and heart rate variability in female university students: a randomised cross-over trial

**DOI:** 10.3389/fnut.2025.1586409

**Published:** 2025-09-19

**Authors:** Sanjoy K. Deb, Nadia Kim, Brenda Parolin, Derek Renshaw, Mohammed Gulrez Zariwala

**Affiliations:** ^1^Centre for Nutraceuticals, University of Westminster, London, United Kingdom; ^2^Centre for Better Living, Anglia Ruskin University, Cambridge, United Kingdom; ^3^Centre for Health and Life Sciences (CHLS), Coventry University, Coventry, United Kingdom

**Keywords:** palmitoylethanolamide, stress, heart rate variability, university students, autonomic regulation, salivary cortisol, mood

## Abstract

**Background:**

Chronic stress is a prevalent issue among university students, negatively affecting both mental and physiological health. Palmitoylethanolamide (PEA), particularly in the Levagen+® formulation, has been investigated for its potential stress-modulating effects through its anti-inflammatory and neuroprotective properties. This study aimed to assess the effects of 6 weeks of Levagen+® PEA supplementation on physiological and subjective markers of stress in moderately stressed female university students.

**Methods:**

A double-blind, placebo-controlled crossover trial was conducted with 16 female participants who met the inclusion criteria based on the Perceived Stress Scale (PSS). Participants were randomly assigned to receive either 6 weeks of PEA supplementation (600 mg/day) or a placebo, with a six-week washout period. Stress responses were assessed through heart rate variability (HRV), subjective stress and mood measures (PSS, PANAS), and salivary cortisol levels. To enhance ecological validity, assessments were conducted in real-life settings rather than laboratory environments.

**Results:**

PEA supplementation significantly increased the Standard Deviation of Normal-to-Normal (SDNN), a key HRV marker associated with autonomic resilience to stress (+9.70 ± 6.02 ms) compared to placebo (−5.72 ± 3.14 ms, *p* = 0.024), suggesting enhanced physiological stress regulation. While there was a trend of increased Root Mean Square Successive Difference (RMSSD) with PEA, it did not significantly change between conditions (*p* = 0.087). Similarly, a trend toward reduced self-reported stress was observed, though it did not reach statistical significance. No significant changes were detected in positive (*p* = 0.78) or negative (*p* = 0.95) emotions experienced. Salivary cortisol levels remained unchanged between conditions (*p* = 0.70).

**Conclusion:**

This exploratory study demonstrates for the first time that PEA supplementation may enhance physiological resilience to stress as indicated by improved HRV. While subjective stress and emotional measures did not show significant changes, the observed trend suggests potential benefits in individuals experiencing moderate stress. Given PEA’s role in the endocannabinoid system and its influence on inflammation, further research is warranted in larger and more diverse populations, including individuals with higher baseline stress levels. These preliminary findings contribute to the growing body of evidence supporting PEA as a promising dietary intervention for stress management.

**Clinical trial registration:**

https://clinicaltrials.gov, NCT06225440.

## Introduction

The human body is intricately programmed to maintain a predefined steady state fundamental to normal life and well-being, termed homeostasis. This state of optimal equilibrium is normally maintained through an elegant interplay between various physiological systems despite constant intrinsic and extrinsic challenges described as stressors (1). Although stress is a broad term not commonly described by a consensus definition, in the biological sense, it is defined as a maladaptive state of threatened homeostasis triggered by stressors that may be physical, psychological, behavioural, or combinations of these (1). A complex network involving the hypothalamic–pituitary–adrenal (HPA) axis and the autonomic nervous system (ANS) acting in concert with the key centres in the central nervous system (CNS) and peripheral tissues are all mobilised to generate an adaptive, coping response to stress. Acute stress arises from immediate challenges or stressors with the body mobilising the classic ‘fight or flight’ responses mediated by sympathetic nervous system (SNS) activation. This initiates a cascade of hormonal secretions including catecholamine release, and subsequent physiological changes such as increased arterial pressure and blood flow to muscles (2). Mobilisation of acute responses does not necessarily place a health burden on an individual and provides positive or rewarding stimuli (3). However, prolonged exposure to stressors and sustained mobilisation of adaptive stress responses can lead to maladaptive physiological function and physical, psychological and behavioural impairments contributing to disease (4). For example, psychosocial stress has been shown to be a significant risk factor in the development of anxiety disorders, a major health concern globally and one of the most diagnosed categories of mental health (5). Several studies have shown sex differences in stress responses, for example, in an academic context, research has identified a gender gap, with females more likely to experience greater levels of stress (6, 7). These effects may be compounded by lifestyle choices, including adherence to a Western-style diet, which has been linked to elevated stress levels in female college students, potentially due to its pro-inflammatory properties (8). Consequently, interventions aimed at reducing inflammation may help mitigate stress within this population (9).

Palmitoylethanolamide (N -palmitoylethanolamine or PEA) is an endogenous fatty acid amide belonging to the family of N-acylethanolamines (NAEs) that is ubiquitously produced in the body (10). NAEs include the endogenous cannabinoid receptor ligand anandamide (AEA, arachidonoylethanolamide) and the satiety agent oleoylethanolamide (OEA). However, despite structural similarities, PEA does not act as a classical endocannabinoid and has a rather complex and varied pharmacological profile (11). Originally referred to as ALIAamide (ALIA - autacoid local inflammation antagonist) on the basis of observations that it reduced mast cell degranulation, PEA has since been shown to interact with a range of receptor and non-receptor targets to produce a host of biological effects, including anti-inflammatory, analgesic, antiviral and neuroprotective (10). PEA’s most well-documented actions are mediated via the nuclear transcription factor peroxisome proliferator-activated receptor alpha (PPAR-*α*), via which it is shown to exert anti-inflammatory actions (12, 13). Although not a direct ligand of the cannabinoid receptors (CB1 and CB2), PEA has been shown to act indirectly by potentiating the actions of endocannabinoids such as AEA via an ‘entourage effect’ (13, 14). In this model, PEA increases receptor affinity for AEA by reducing its enzymatic degradation via the enzyme fatty acid amide hydroxylase (FAAH) by serving as an alternative substrate, thereby enhancing its effects attributed to AEA (13).

In addition to its endogenous expression, PEA is also available exogenously through the diet and is present in various foods such as soy lecithin, palm oil, peanut meal and egg yolk. However, the available levels are relatively low, giving rise to the utilisation of exogenous PEA in the form of dietary supplements. However, issues persist with exogenous PEA administration due to its poor pharmacokinetic profile, which leads to the application of strategies such as micronisation and carrier-based delivery systems to enhance its bioaccesibility (11). Levagen+® is a formulated form of PEA utilising a cold-water dispersible (CWD) technology (LipiSperse®) previously shown to have enhanced bioavailability (15). Recent clinical studies have shown Levagen+® PEA to improve sleep quality as well as parameters of cognitive function (16, 17). Given the close interplay between these functionalities, the observed beneficial effects of supplemental PEA on markers of wellbeing and cognition raises intriguing possibilities for exploring its role in mitigating stress. Based on this rationale, the current study aimed to address the research question: Does 6 weeks of supplementation with Levagen+® (a formulated PEA) improve physiological and subjective markers of stress in moderately stressed female university students compared to placebo?

## Methods

### Study design

A randomised, double-blinded, placebo-controlled cross-over approach was utilised to examine the impact of PEA supplementation on stress, mood and heart rate variability in university students. This approach was selected to reduce selection and allocation biases, control for potential confounding factors, and ensure that every participant received both PEA supplementation and a placebo in a counterbalanced order. Participants were asked to self-record measurements at home on four occasions across two academic semesters, with follow-up assessments scheduled during periods of assignments and exams to capture periods that have been shown to elicit stress in university student populations (18). The day before the collection period, participants attended the laboratory and were given an at-home salivary collection kit and fitted with a heart rate variability monitor (described below). HRV, morning salivary cortisol, and subjective measures (Perceived Stress Scale and PANAS) were all recorded during the first hour after waking, on the morning following the laboratory visit. Twenty-four hours before collection, participants were asked to abstain from alcohol and stimulant consumption, extensive training, sauna use, sleep deficit, exposure to excessive noise and illuminance and maintain hydration status to minimise potential confounding factors. The manuscript was written in accordance with the Consolidated Standards of Reporting Trials (CONSORT).

### Participants

A subset of female participants from a larger randomised controlled trial (ClinicalTrials.gov Identifier: NCT06225440) were invited to take part in this exploratory sub-study. Selection was based on scoring in the moderate range of perceived stress (PSS > 13), as this population has not been specifically investigated in the context of PEA supplementation. In addition, due to logistical considerations, only a defined number of participants could be provided with validated HRV monitoring devices capable of capturing reliable morning recordings. This approach allowed for a focused analysis of HRV responses in a moderately stressed cohort while maintaining feasibility within the operational constraints of the larger study.

**Figure 1:**
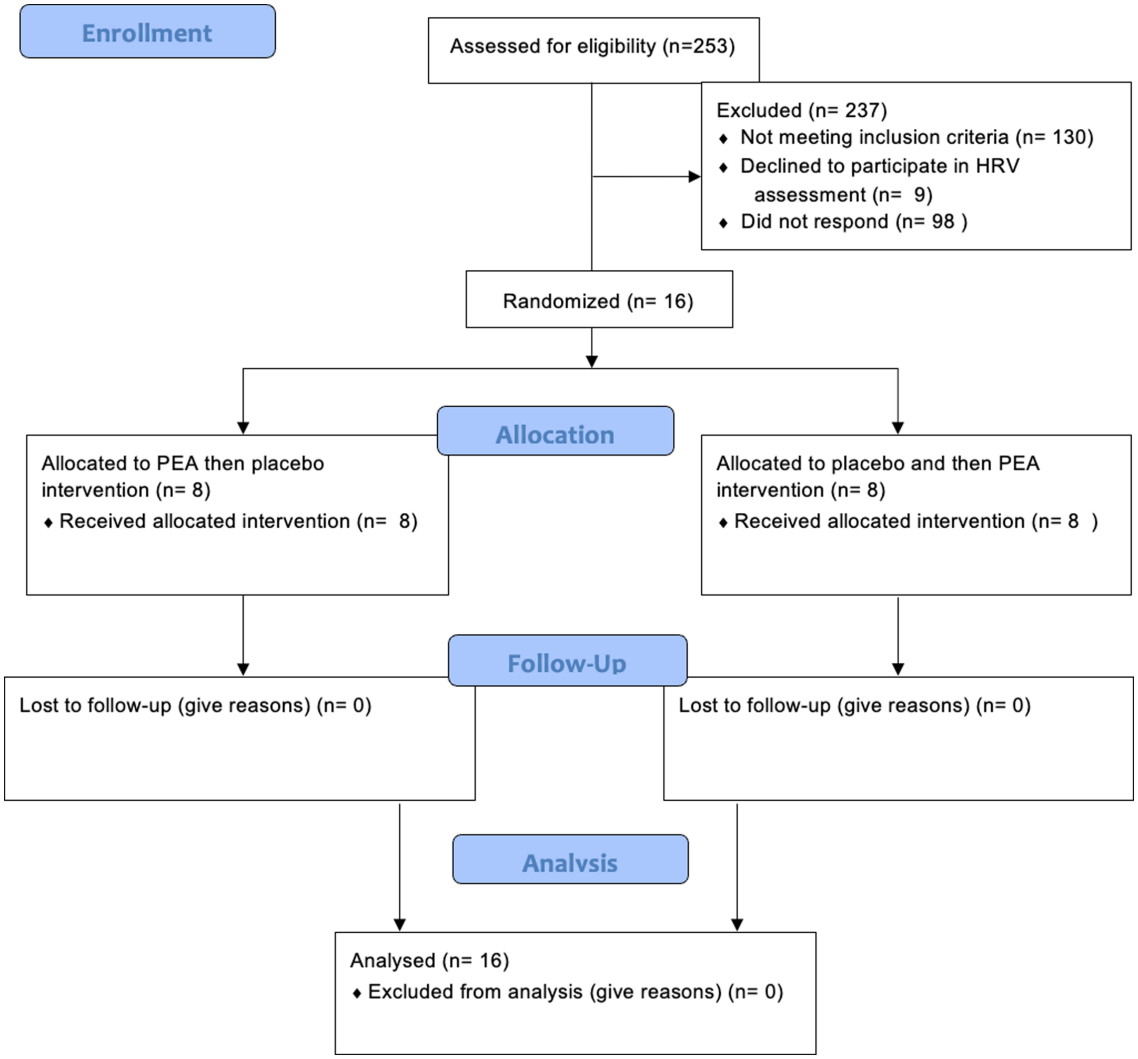
Participant recruitment flow diagram.

Sixteen female participants were recruited for this part of the study (mean ± SD age: 22 ± 2.4 years; Figure 1). They were required to be fully enrolled in their courses and committed to completing the entire intervention. Female participants who reported being moderately stressed were recruited, as determined by the Perceived Stress Scale (PSS; described below). Individuals with a score greater than 13 were determined to have moderate stress levels (19). Moderately stressed individuals (PSS > 13) were selected to reflect a non-clinical population that may benefit from early lifestyle or nutraceutical interventions before progression to chronic stress-related disorders. This approach also aligns with ethical considerations of conducting supplementation trials in otherwise healthy participants and avoids confounding effects related to pharmacological treatment or psychiatric comorbidities commonly seen in severely stressed or clinical populations. Additionally, recruiting moderately stressed individuals improves generalisability to broader populations, such as university students, where subclinical stress is highly prevalent.

The exclusion criteria comprised individuals with chronic health conditions, learning disabilities, smoking, and excessive alcohol consumption (more than 14 units per week), as well as individuals with neurological disorders or on medication, including those using dietary supplements and herbal remedies, were excluded from participation. Additional exclusions included individuals who are obese, pregnant, breastfeeding, attempting conception, or undergoing/planning medical, dental, or orthodontic procedures. Online recruitment via social media was used to target students in higher education at any local London university. Interested volunteers completed a survey to screen against the eligibility criteria. The first on-site visit involved measuring participants’ body mass index (BMI) and blood pressure, with eligibility limited to those having a BMI between 20 and 30 and blood pressure below 140/90 mmHg. Prospective participants were briefed on the study, which included reviewing the Participant Information Sheet and detailing the study’s objectives, procedures, and potential risks and benefits. Following this detailed explanation, each participant provided a signed consent form, signifying their willingness to partake in the study. The faculty ethics committee at the University of Westminster granted ethical approval for the study (Application ID: ETH2122-1031).

### Intervention

Participants were randomly assigned to one of two treatment sequences in a double-blind, placebo-controlled crossover design: (1) Levagen+® (formulated PEA) followed by placebo or (2) Placebo followed by Levagen+®. Randomisation was conducted using an unrestricted probability sampling approach via a computer-generated sequence,^1^ ensuring equal allocation across conditions. Each treatment phase lasted 6 weeks, separated by a two-week washout period to minimise carryover effects. This design is consistent with previous crossover trials involving PEA supplementation (15, 16).

Participants received daily doses of either Levagen+® (Gencor Pacific Limited, Lantau Island, Hong Kong) or placebo (microcrystalline cellulose). The active supplement (Levagen+®) delivered 600 mg of PEA per day (two capsules containing not less than 300 mg PEA each) using LipiSperse® dispersion technology (Pharmako Biotechnologies Pty Ltd., Sydney, Australia) to enhance bioavailability. Capsules in both conditions were identical in size, shape, and colour, and were manufactured by Power Health Products Ltd. (York, United Kingdom). Participants were instructed to take both capsules simultaneously each day for the six-week period. Regular researcher contact helped support adherence, and no adverse effects were reported.

The selected dose of 600 mg/day of palmitoylethanolamide (PEA) in the present study is based on the bioavailability and efficacy profile of the formulation used—Levagen+®, which incorporates LipiSperse®, a delivery system that enhances water dispersion and has been shown to improve PEA bioavailability and uptake in humans (15). Several studies have demonstrated favourable outcomes across a range of clinical applications and dosages. For example, 300 mg/day of Levagen+® over 8 weeks significantly reduced sleep onset latency and improved next-day cognition (17). A daily dose of 450 mg/day was associated with a reduction in headache duration and faster resolution of severe headaches compared to 400 mg/day of ibuprofen (20). Notably, 600 mg/day of Levagen+® led to greater headache resolution at 2 and 8 h, lower pain scores at 1.5 and 4 h, and reduced reliance on rescue medication in individuals with recurrent migraines, compared to placebo (21). In the context of mental health, 600 mg/day over 8 weeks also resulted in significantly reduced symptoms of depression and anxiety (22). Taken together, these findings support the selection of 600 mg/day for the current study, aligning with prior evidence on both the safety and efficacy of this dose and its relevance to the targeted outcome measures.

### Heart rate variability

Short-term HRV was recorded and analysed at home during a 5-min rest period on waking in the morning. Short-term HRV measurement is the most commonly used assessment of HRV (23) and is posited to be a useful biomarker to assess the effect of dietary interventions on physical and mental health (24). Participants in the study were equipped with The Firstbeat Bodyguard 2 (BG2, Firstbeat Technologies Oy, Finland), a sensor designed for short and long-term HRV measurements. This lightweight (Weight: 24 grams, Dimensions: 47 mm x 63 mm x 10.6 mm) and easy-to-use device, affixed directly to the skin with two chest electrodes, automatically initiated data recording. The researcher demonstrated correct application in person, and further written instructions were provided at home. Participants were asked to wear the HRV upon waking so that initial resting measurements could be taken. Data obtained from the Firstbeat Bodyguard 2 (BG2) devices were extracted using the offline mode of the Firstbeat uploader software (version 3.4.4.0). Subsequently, the extracted data were imported into Kubios HRV Scientific (version 4.1.0). Kubios was used to determine HRV parameters, encompassing time and frequency domain measures. Time domain measures included standard deviation of normal-to-normal intervals (SDNN), root mean square of successive differences (RMSSD), and the proportion of adjacent RR intervals differing by more than 50 ms (pNN50). Frequency domain measures of low-frequency (LF) and high-frequency (HF) ratios were also analysed.

### Subjective assessment of stress and mood

The Perceived Stress Scale (PSS) was used to monitor stress at the beginning and end of each experimental arm. The PSS has been widely used to assess the effect of dietary interventions on stress (25–27) and is a valid tool to assess stress in a university study cohort (28). Similarly, the Positive and Negative Affect Schedule (PANAS) was completed pre- and post-experimental treatment arms (29). The Positive and Negative Affect Schedule (PANAS) is a widely used 20-item scale that measures positive and negative emotions through two distinct subscales. The Positive Affect (PA) scale assesses feelings of engagement and enthusiasm, while the Negative Affect (NA) scale captures distressing emotions such as fear and anger. The PANAS has demonstrated strong psychometric properties, including excellent reliability (*α* = 0.89 for PA and α = 0.85 for NA) (30).

### Salivary cortisol collection and analysis

Saliva samples were collected using a non-invasive method that involved the participant inserting a Sarstedt Salivette® (Sarstedt, Germany) cotton bud containing a saliva preservative inside their mouth for 2 min. To ensure appropriate collection techniques, participants completed a supervised collection during each visit and were asked to replicate this the following day on waking (between 7 and 8 am). Participants were instructed to keep the collected samples in their home fridge before returning them to the laboratory, where they were aliquoted and stored at −80°C until analysis. Cortisol levels were determined using a competitive enzyme-linked immunosorbent assay kit (Salimetrics, Carlsbad, CA).

### Statistical analysis

As initial assumptions and tests of normality were met, a paired *t*-test was used to compare the change score of all outcome variables between baseline and follow-up for the placebo and PEA conditions. A significance level of *p* < 0.05 was applied to determine statistical significance. All analysis was conducted on SPSS 19 (IBM, Chicago, IL, United States) and Microsoft Excel (Microsoft, Redmond, WA, United States). All descriptive data are reported as mean and SEM.

## Results

### Heart rate variability measurement

**Figure 2:**
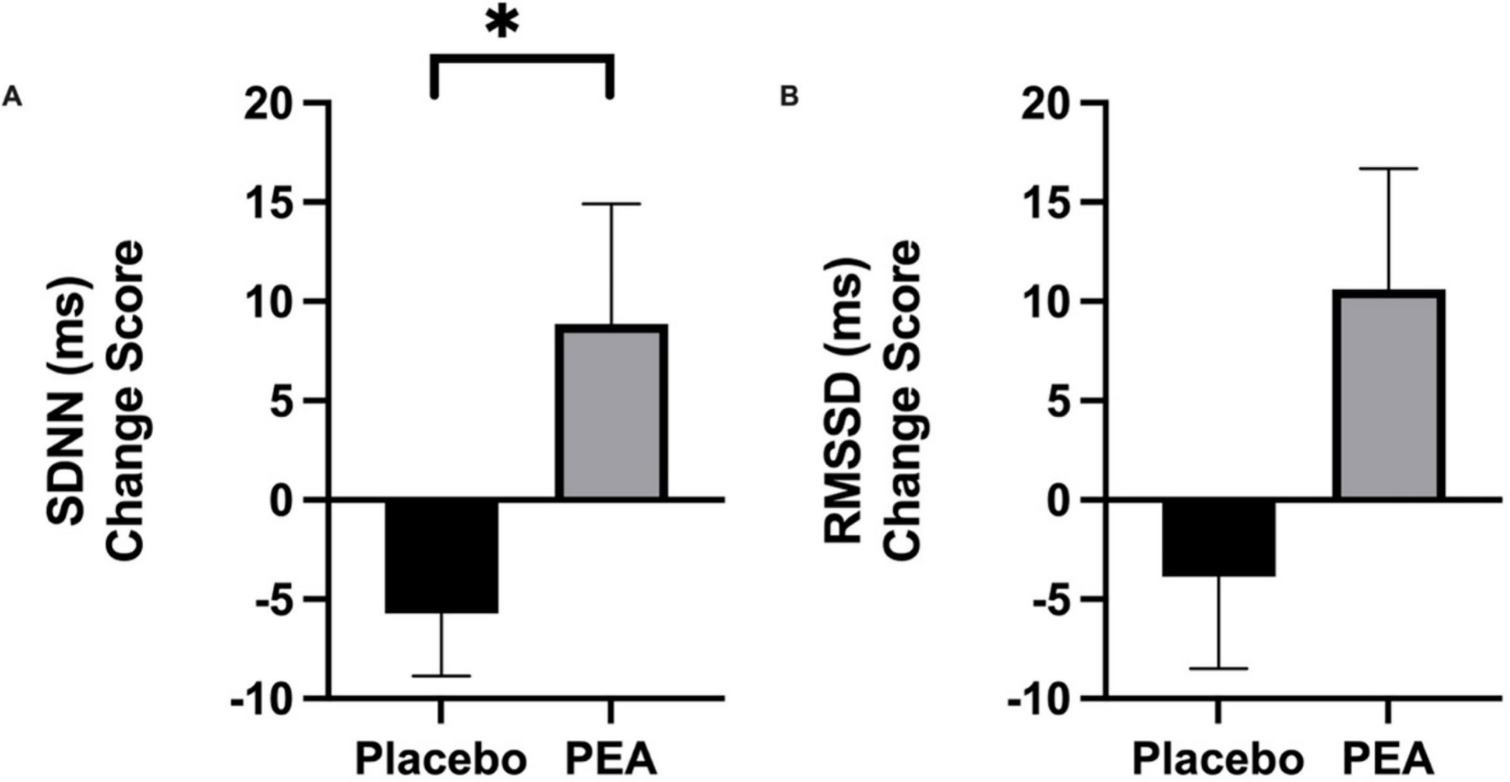
Intervention pre-post change scores comparing placebo and PEA Levagen+® for HRV variable **(a)** Standard Deviation of Normal-to-Normal (SDNN) and **(b)** Root mean square of successive differences (RMSSD). *represent statiscally signifcant difference *p* < 0.05.

Six weeks of supplementation with Levagen+® PEA significantly affected SDNN (Figure 1). SDNN saw a significant increase of 9.70 ± 6.01 ms in the PEA treatment arm compared to a reduction of −5.72 ± 3.14 ms in the placebo arm (*p* = 0.024). Although there was a trend for an increase in other time domain measurement of RMSDD with PEA (+10.60 ± 6.08 ms) compared to placebo (−3.86 ± 4.62 ms; *p* = 0.087; Figure 2). However, there were no differences in the low-to-high frequency ratio between placebo and PEA (*p* = 0.965). Furthermore, no order effect was found across these HRV variables (*p* > 0.05).

### Subjective stress and mood

**Figure 3:**
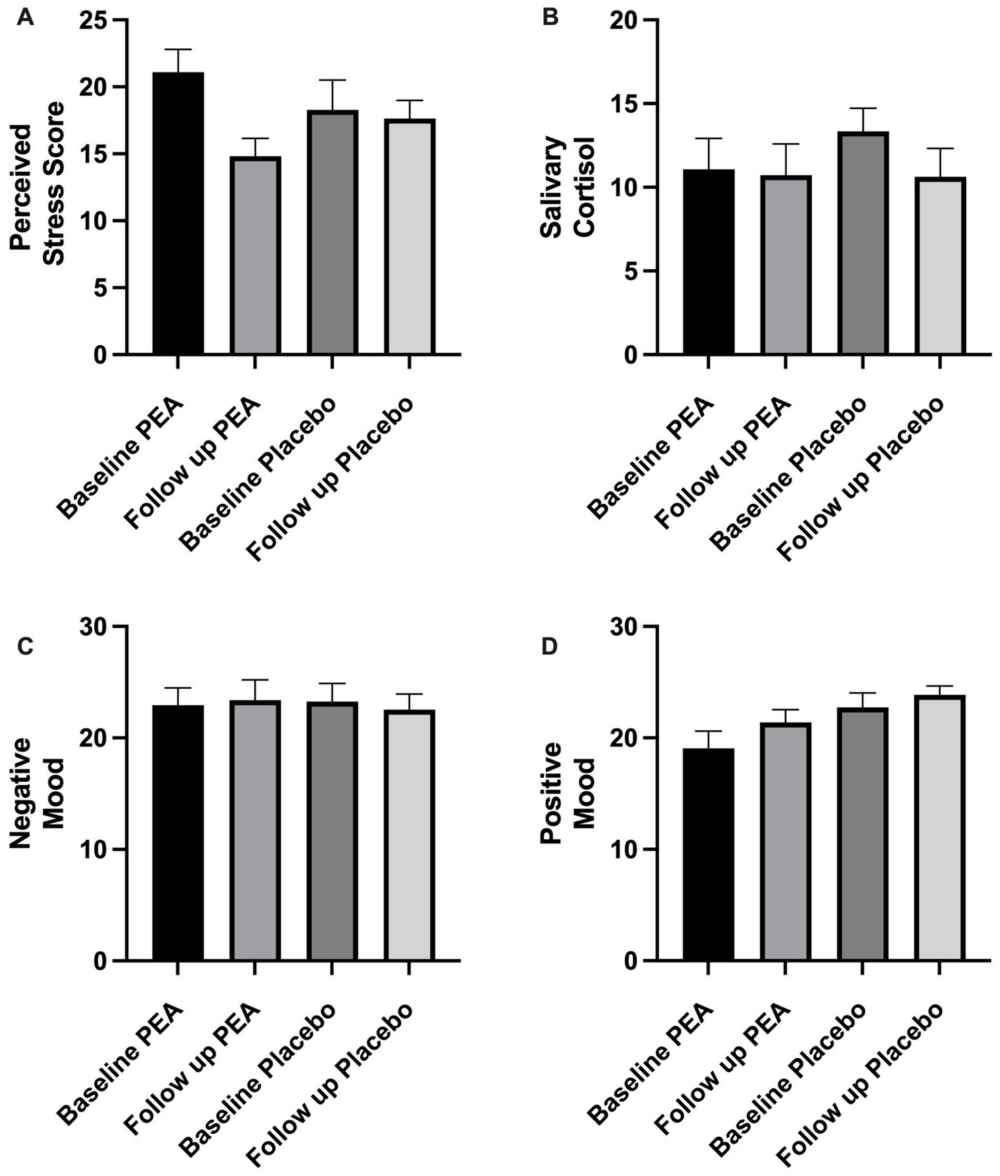
Showing pre and post-intervention comparing placebo and PEA Levagen+® for **(a)** perceived stress score, **(b)** salivary cortisol, **(c)** negative mood and **(d)** positive mood. No significant differences were observed between conditions.

At the start of both treatment arms, participants were moderately stressed, averaging 21.09 ± 1.70 (PEA) and 18.27 ± 2.23 (placebo). While there was a trend in the reduction following PEA supplementation (14.82 ± 1.32) compared to placebo (17.64 ± 1.35), there were no significant differences between conditions (*p* = 0.10; Figure 3). Equally, no difference in positive (*p* = 0.78) or negative (*p* = 0.95) emotions was observed between the two conditions (Figure 3). No order effect was found across stress and mood variables (*p* > 0.05).

### Salivary cortisol

Salivary cortisol values did not change between PEA and placebo treatment arms. Baseline values were 11.07 ± 1.84 nmoL/L and 13.35 ± 1.37 nmoL/L, which were not significantly different at the end of the intervention period, with values of 10.73 ± 1.86 nmoL/L and 10.63 ± 1.69 nmoL/L for PEA (*p* = 0.64) and placebo (*p* = 0.41), respectively (Figure 3). Change scores between PEA (−1.06 ± 0.97 nmoL/L) and placebo (−2.72 ± 2.13) did not differ (*p* = 0.49). No order effect was found for salivary cortisol (*p* > 0.05).

## Discussion

This study aimed to assess the effects of 6 weeks of Levagen+® formulated PEA supplementation in moderately stressed female participants on markers of objective physiological stress (HRV and salivary cortisol) and subjective reports of stress and emotions. Using a double-blind, randomised controlled trial, participants were asked to report markers of stress on waking at the participant’s home to give the study a higher degree of ecological validity. This meant participants were not exposed to artificial acute stress from laboratory visits, which is seldom experienced in daily life. The study found an increase in the HRV parameter of SDNN and a trend toward reducing self-reported stress. Measurements of morning salivary cortisol and self-reported emotions did not change. As such, this study demonstrates the potential of PEA to mitigate physiological stress in female university students, and it may be a plausible dietary intervention in moderately stressed individuals.

Heart rate variability has been posited as a biomarker to assess a nutritional intervention’s physiological and psychological effects due to the consistent relationship reported between HRV and health outcomes (24). In accordance with this, the main finding from the study was the reduction in SDNN following 6 weeks of Levagen+® PEA supplementation. This aligns with previous research that has demonstrated the influence of dietary interventions in increasing SDNN measurements following omega-3 (31, 32) and polyphenol-rich treatment (33). Like these nutrients, PEA possesses anti-inflammatory properties that may explain the effect on HRV (34). Indeed, there is a negative correlation between C-reactive protein (CRP), a marker that indicates pro-inflammatory conditions, and SDNN (35), suggesting PEA may contribute to lower levels of inflammation and result in a higher SDNN. High-frequency (HF) components of HRV are also linked with CRP, with reports that lower HF-HRV predicted a higher CRP 4 years later (36). The current study did not observe a link between HF-HRV and PEA supplementation. However, this may be due to the short-term intervention used, and extended intervention periods may be required to determine whether PEA has benefits for HRV. It should be acknowledged that an unexpected reduction in both SDNN and RMSSD was observed in the placebo condition. This may be attributed to elevated stress during the follow-up assessment period, which coincided with academic deadlines. While this aligns with previous evidence showing HRV is impacted by stress (37), the absence of this decline in the PEA group was a novel finding in the current study. The increased SDNN observed may suggest cardiovascular benefits, with a meta-analysis reporting that a 1% increase in SDNN is associated with a 1% risk reduction in cardiovascular disease risk (38).

Despite improvements in HRV parameters in a self-reported stressed cohort of female students, we did not find any significant effect on the subjective reports following PEA supplementation. SDNN is suggested to be an index of physiological reserve against stress (37), which may explain the trend toward reduced stress following PEA. Previous research has reported a link between SDNN and self-reported work stress, with greater stress associated with a lower SDNN (39, 40). Equally, in university students, the stress caused during examination periods was found to have a lower SDNN (41). The observations in the current study do not align with this theory, as the increased SDNN following PEA supplementation did not result in significantly lower self-reported stress. It should be noted that larger-scale ecological studies conducted with workers *in situ* suggested that the significant association between SDNN and stress was small (42). As such, the smaller sample size in the current study may contribute to the lack of significance observed in subject stress.

This study also reported no differences in positive or negative emotions with the PEA intervention. This is in contrast with previous research that reported 6 weeks of PEA supplementation has been shown to reduce depression in patients with major depressive disorder (43), which is also supported in pre-clinical mice models (44). In a retrospective analysis of COVID-19 patients, it was reported that those taking PEA reported fewer depressive symptoms, with authors suggesting that individuals who were identified to have pre-treatment fatigue or report poor subjective well-being were more likely to benefit from a PEA intervention (45). Furthermore, recent reviews have proposed that plasma PEA may emerge as a biomarker for psychosis, and supplementation could be seen as a therapeutic treatment (46). Therefore, it is likely that the stress and low emotions experienced in the sample recruited for this study were not severe enough to report improvements in subjective mental health parameters. There remained a plausible effect from PEA in the general population, and further research may consider PEA interventions on individuals who report high-stress levels and depressive symptoms, which are classed as sub-clinical.

Salivary cortisol did not respond to PEA intervention in the current study, which aligns with previous research assessing dietary intervention. While there have been examples of anti-inflammatory supplements reducing salivary cortisol, such as dark chocolate polyphenols and omega-3 (47–49), much of the research has shown a high degree of variability (50). Pre-clinical mice models have suggested that PEA may act on the HPA axis by reducing the expression of hypothalamic corticotropin-releasing hormone and its type 1 receptor (51). This hormone is the primary driver of the body’s stress response, so there remains promise for PEA. Cortisol measurements demonstrate widespread diurnal variability (52), rendering it challenging to determine an intervention effect. This may explain the inconsistent findings across nutritional intervention research (50) and that observed in the current study. Future investigations may consider a more comprehensive assessment of cortisol response, including multiple daily measurements and assessments following acute artificial stress in laboratory settings.

The endocannabinoid system (ECS) is central in modulating stress reactions and adaptations to chronic stress via the recruitment of the endocannabinoid AEA and the related NAEs PEA and OEA (53). It regulates cardiovascular function, including heart rate and blood pressure, which are key elements of HRV, with AEA implicated as the key actor (W-SV Ho). Indeed, a recent study examining endocannabinoid-HRV interactions in response to stress reported that AEA, PEA and OEA were noticeably increased in hair samples of individuals partaking in intense, prolonged exercise (54). Taken together, these observations support a model wherein AEA and PEA may play a central role in adaptive responses to stress via modulation of HRV, and in this scenario, supplementation with bioavailable, exogenous PEA would further sustain and amplify these effects as observed in this study.

The conclusion drawn from this study should be viewed in the context of certain limitations. Due to logistical challenges, the sample from this study was recruited from a subset of a larger study (17). *Post Hoc* analysis shows the power of the primary outcome was 57%; however, previous studies examining the positive effects of dietary interventions on HRV use similar sample sizes (55, 56). While the smaller sample size may increase the risk of a Type II error, the findings from this study provide early insights into the role of PEA in modulating autonomic function. The lack of effect on secondary variables from the PSS and PANAS shows a disconnect between physiological HRV measurements and subjective markers. It is likely that these perceptual variables are not sensitive enough to detect changes within the current sample size. Alternative scales should also be considered, such as the State–Trait Anxiety Inventory (STAI), to capture nuanced emotions. Future studies could use Ecological Momentary Assessment (EMA) to overcome the limitations of retrospective mood and stress assessments, which can be confounded by daily stressors and the dynamic fluctuations of emotional states. The use of at-home assessments was a novel aspect of this study, enhancing ecological validity; however, unsupervised data collection introduces potential variability due to environmental factors and participant compliance. To mitigate these concerns, participants received detailed instructions, supervised practice sessions, written guidance, and regular check-ins by researchers throughout the study. Furthermore, the present study intentionally focused on female participants due to their greater vulnerability to stress-related disorders; the absence of male participants limits the generalizability of our findings. To further clarify potential sex-specific responses to PEA supplementation, future research should incorporate male comparator groups. Furthermore, menstrual cycle phase and hormonal contraceptive use were not recorded in this study. While these factors can influence salivary cortisol concentrations, HRV has been shown to be relatively unaffected across the menstrual cycle (57). Nevertheless, we acknowledge this as a limitation in interpreting endocrine-related measures and recommend that future studies account for reproductive hormone status. Lastly, several potential confounding factors such as acute daily stressors, academic workload fluctuations, social support, and socioeconomic status were not systematically controlled or assessed in this study. These variables are known to influence both subjective and physiological stress responses and may have contributed to the variability and limited number of significant findings. Although the study design aimed to enhance ecological validity by assessing participants in their usual environments, this approach also introduced variation in daily experiences that could not be fully accounted for. Future studies should consider including contextual data collection methods, such as daily diaries, EMA, or indicators of socioeconomic background, to better control for these influences.

To better address these limitations, future studies could integrate real-time, continuous measures such as EMA for subjective stress, multiple diurnal cortisol samplings, and laboratory-based stress induction procedures. Additionally, assessments of inflammatory biomarkers (e.g., C-reactive protein, interleukin-6) should be included to elucidate potential underlying mechanisms linking PEA supplementation with observed HRV improvements. Alternatively, laboratory-based studies could introduce acute stressors previously validated to induce physiological and psychological stress responses measurable via HRV. Employing such controlled protocols would allow for a clearer understanding of how physiological, biochemical, and subjective stress responses interact and respond to nutraceutical interventions like PEA supplementation.

## Conclusion

PEA has been relatively well studied in the context of inflammation and immune health; however, its putative benefits on stress and cognition have been explored less. This preliminary study evaluated the effects of PEA supplementation on markers of stress in females moderately stressed at onset, demonstrating for the first time an increase in the HRV parameter of SDNN and a trend toward reducing self-reported stress. HRV assessments indicate stress in various situations and are frequently used as a sensitive digital assessment tool in this regard, with SDNN regarded as an index of physiological resilience against stress (58). Our data, therefore, provide a meaningful rationale for using PEA supplementation in stress management. The sample size and the specificity of the study’s inclusion criteria may have contributed to the lack of significance observed in other measures; the work, therefore, also serves as a foundation for more comprehensive examinations to further clarify the findings.

## Data Availability

The raw data supporting the conclusions of this article will be made available by the authors, without undue reservation.

## Footnotes

^1^ https://www.graphpad.com/quickcalcs/randomize1/
